# Exophytic benign mixed epithelial stromal tumour of the kidney: case report of a rare tumour entity

**DOI:** 10.1186/1746-1596-5-16

**Published:** 2010-03-01

**Authors:** Michael Richter, Werner Meyer, Jens Küster, Peter Middel

**Affiliations:** 1 Department of Urology, Nephrologisches Zentrum Niedersachsen, Am Vogelsang 105, 34346 Hann-Münden, Germany; 2 Institut for Pathology, Pathologie Nordhessen, Wilhelmshöher Allee 287, 34131 Kassel, Germany; 3 University of Göttingen, KFO 193, Robert-Koch-Strasse 40, 37075 Göttingen, Germany

## Abstract

**Background:**

Mixed epithelial and stromal tumour (MEST) represents a recently described benign composite neoplasm of the kidney, which predominantly affects perimenopausal females. Most tumours are benign, although rare malignant cases have been observed.

**Case report:**

A 47-year-old postmenopausal female presented to the urologist with flank pain. A CT scan of the abdomen showed a 30-mm-in-diameter uniform mass adjacent to the pelvis of the left kidney. Surgical exploration showed a tumour arising from the lower anterior hilus of the left kidney. The tumour could be excised by preserving the kidney. By intraoperative frozen section the tumour showed characteristic features of MEST with epithelial-covered cysts embedded in an "ovarian-like" stroma. Additional immunohistochemistry investigations showed expression for hormone receptors by the stromal component of the tumour.

**Discussion:**

MEST typically presents in perimenopausal women as a primarily cystic mass. Commonly, the tumour arises from the renal parenchyma or pelvis. The tumour is composed of an admixture of cystic and sometimes more solid areas. The stromal cells typically demonstrate an ovarian-type stroma showing expression for the estrogen and progesterone receptors.

**Conclusion:**

MEST represents a distinctive benign tumour entity of the kidney, which affects perimenopausal woman. The tumour should be distinguished from other cystic renal neoplasms. By imaging studies it is difficult to distinguish between a benign or malignant nature of the tumour. Thus, intraoperative frozen section is necessary for conservative surgery, since the overall prognosis is favourable and renal function can be preserved in most cases.

## Background

The mixed epithelial and stromal tumour (MEST) of the kidney represents a recently described benign tumour, which has to be distinguished from other renal neoplasms. The term MEST was first introduced by Michal and Syucek in 1998[1]. Morphologically, the tumour is characterized by a biphasic proliferation of stromal cells with an epithelial component showing cystic dilatation. The vast majority of cases show a benign course without tumour recurrence [2-4].

We report the unusual case of a MEST arising from the left kidney as a cystic tumour in an exophytic fashion. Since the diagnosis was secured by intraoperative frozen section, tumour excision without nephrectomy could be performed as treatment of choice.

## Case presentation

**Figure 1:**
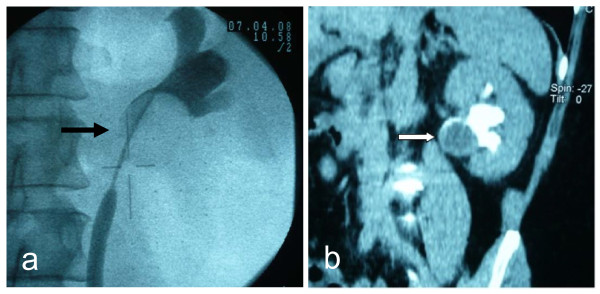
**Retrograde ureterography demonstrated a sharply circumscribed tumour mass (black arrow) leading to obstruction of the proximal left ureter (a)**. Contrast enhanced CT-Scan showed a homogenous mass located adjacent to the lower hilus of the left kidney (white arrow) without significant contrast enhancement **(b)**.

A 47-year-old postmenopausal female patient presented to us with increasing left-sided flank pain. Physical examination was unremarkable. Aside from a well-medicinally treated hypertension no accompanying disease was present. Her routine blood investigations were normal. Urine cytology revealed suspicions of atypical urothelial cell elements. Retrograde ureterography showed an obstruction of the proximal left ureter due to a tumour projecting to the lower-left renal pelvis (Figure 1a). A contrast-enhanced computerized tomographic scan of the abdomen and pelvis showed a 30 × 25-mm hypodense, well-defined mass adjacent to the hilar side of the lower pole of the left kidney with minimal contrast enhancement (Figure 1b). Because of renal obstruction a stent was inserted into the left ureter. There was no evidence of lymph node or distant metastases.

**Figure 2:**
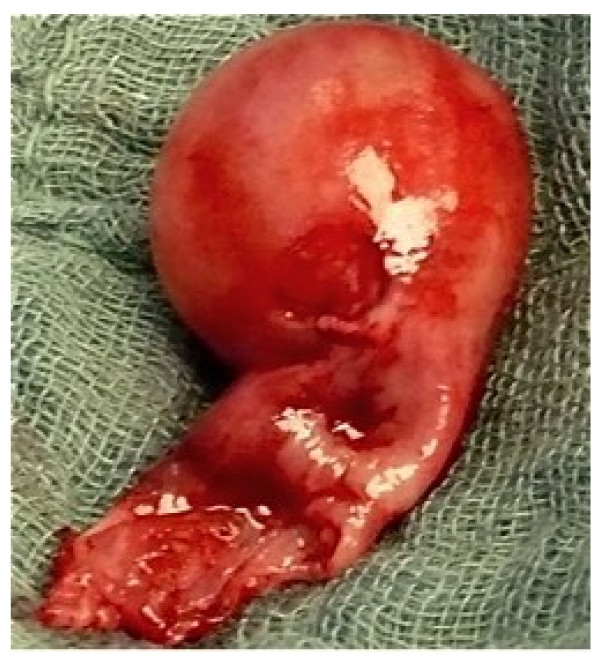
**Surgical specimen**. Upon removal the mass revealed a well defined tumour originating with a narrow pedicle from the papilla of the lower chalice group.

On surgical exploration there was a well-circumscribed tumour mass arising from the lower anterior pole of the left kidney attached with a narrow pedicle to the papilla of the lower chalice group (Figure 2). The tumour could be excised completely. Frozen section revealed a solid and cystic tumour of probably benign nature, most probably MEST. After insertion of a double-J stent into the left ureter the renal pelvis was closed. No post-operative complications were observed.

## Results

### Pathologic findings

**Figure 3:**
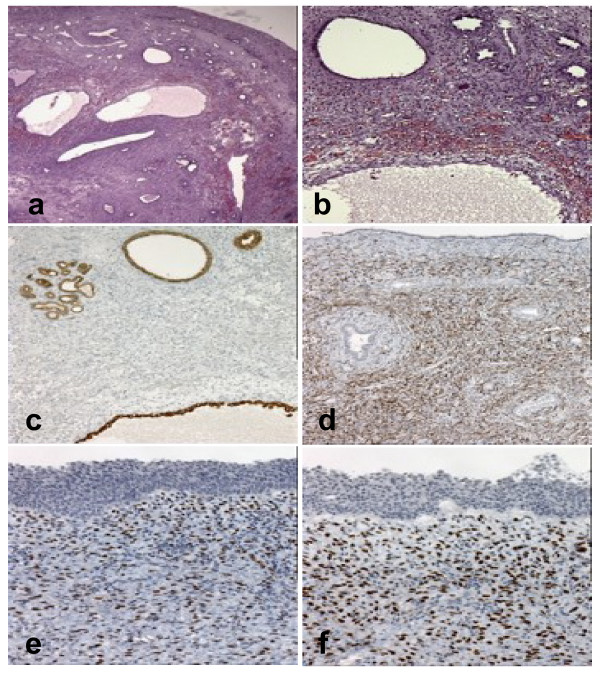
**Low magnification shows a tumour composed of large cysts as well as microcysts, and tubules embedded in a fibrous stroma (A, H&E, ×40)**. Focally, an "ovarian-like" stroma was observed (B, H&E, ×100). The epithelial component demonstrated expression for cytokeratin-7 (C, ×100), whereas the "ovarian-like" stroma showed coexpression for smooth-muscle actin (D, ×100) as well as for estrogen (E, ×100) and progesterone receptors (F, ×100).

Gross examination showed a 2.5 × 2 × 1.5-cm-in-diameter, well-encapsulated partly cystic tumour with a 2-cm-long narrow pedicle. Histologically, the tumour was composed of cysts embedded in a collagenous stroma containing bundles of spindle cell proliferations with variable cellularity. The cysts varied significantly in diameter and were covered by a flat or cuboidal epithelium with areas of clear cell differentiation and hobnail appearance. The thickness of the cyst septi ranged from one to 6 mm microscopically. The relative proportion of the stromal component to the entire tumor (stroma to cyst ratio) was about 55%. No dysplasia, increased mitotic activity or tumour necrosis as signs for malignant transformation were observed. A distinctive feature was focal condensation of stromal cell adjacent to the cysts (Figure 3a and 3b). By immunohistochemical investigations, the epithelial cells demonstrated expression for pan-cytokeratin and cytokeratin-7 (Figure 3c), whereas the stromal cells demonstrated co-expression for mesenchyme markers such as vimentin, desmin, and smooth-muscle (sm) actin (Figure 3d). Focally, expression for CD10 and alpha-inhibin by stromal cells could be observed. Constantly, the latter areas demonstrated moderate to strong nuclear expression for the estrogen- and progesterone-receptors (Figures 3e and 3f), thus resembling a so-called "ovarian-like" stroma. Focally, a weak expression for vimentin was observed by very few epithelial cells of the cysts.

## Discussion

Mixed epithelial and stromal tumour (MEST) represents a recently described tumour entity of the kidney of unknown etiology [2,5,6]. In the past other synonyms, such as cystic harmatoma of the renal pelvis, adult mesoblastic nephroma, and cystic nephroma with "cellular" or "ovarian-type" stroma were applied [2]. About 50 cases have been reported in the literature so far [2,3,5,7,8]. Typically, the tumour presents in perimenopausal woman as a combined solid and cystic tumour mass. The mean age of clinical presentation is about 45 years.

The patients usually present to the urologist with non-specific symptoms, such as flank pain, hematuria, or symptoms primarily suggestive of genitourinary infections. The mean tumour size at primary diagnosis is about 6 cm in diameter [2,5,6]. Different hypothesis have been proposed for its origin. The most popular postulation is based on a disturbed hormonal environment, which is typically observed in perimenopausal women or which may be caused by therapeutically applied female hormones. Thus, hormonal imbalance might induce the proliferation of a misplaced immature or fetal mesenchyme, which harbours the capacity for a dual, epithelial and mesenchymal differentiation [2,5,6]. This theory is driven by the observation of the expression for the estrogen and progesterone receptor within the stromal cells by nearly all cases of MEST [2,5,6]. In addition, the latter hypothesis is supported by a study showing that most of the affected women had a long history of treatment with estrogens. However, the only male patient reported so far had a history of diethylstilbestrol therapy for 7 years, applied because of treatment for prostatic cancer.

Nearly all cases described so far demonstrated a benign course without tumour recurrence. However, four cases of aggressive MEST have been described in the literature so far [9-12]. Three patients with MEST with local recurrence of tumour showing a fatal course have been described. In two patients, the recurrent tumour was composed exclusively of malignant transformed stroma [10]. One case displayed malignant transformation of MEST to a sarcomatoid carcinoma with heterologeous differentiation [9].

The differential diagnoses include renal tumours, which can show at least a partly cystic morphology, such as partially cystic differentiated nephroblastoma, multilocular cystic renal carcinoma, angiomyolipoma with epithelial cysts or in rare cases synovial sarcoma [2,5,6].

MEST demonstrate morphological overlap to cystic nephroma, with which it can be confused [13,14]. Both entities show some remarkable similarities including sex predilection, age distribution, and morphological attributes of the epithelial and stromal components as well as immunohistochemical profile [15]. Albeit there are some variations in the individual categories with a higher prevalence of stromal-to-epithelial ratio, prominent ovarian stroma, smaller cysts more common in MEST; in contrast larger cysts with only thin septa (with septa being smaller than 5 mm) and in addition a lower stromal-to-epithelial ratio are typically observed in cystic nephroma [2]. However, the presence of ovarian-like stroma and müllerian-related immunohistochemical markers raise the possibility that these tumours might originate from müllerian remnants displaced during embryogenesis [2,5,16].

Since some tumours have gross and microscopic features intermediate between cystic nephroma and MEST, it is considered that both tumours might represent different morphological variants of the same tumour entity [2,5-7,14,17,18]. Therefore, Turbiner et al. (2007) proposed to summarize both tumours under the unifying term "renal epithelial and stromal tumour" (REST)[2]. However, this proposal is primarily based on the observation of an ovarian-like stroma in both tumour entities, but since this kind of stroma is also observed in obstructed kidneys, even without neoplasia some authors consider the stromal differentiation as a form of reactive metaplasia [17,19]. In a recent study, Zhou et al. [20] investigated the relationship of cystic nephroma and MEST compared to other renal tumours and normal kidney by using mRNA based gene expression profiling and additional extensive histologic analyses. Unsupervised clustering of mRNA expression profiles demonstrated that cystic nephroma and MEST had very similar expression profiles, which were distinct from other renal neoplasms. Thus, the data of Zhou et al. provide additional convincing molecular evidence that cystic nephroma and MEST represent indeed one disease entity. However, since there are no specific molecular markers for the differentiation of MEST from cystic nephroma and there are no further clues to the origin of both tumour entities, a definitive classification remains outstanding [17].

## Conclusion

Mixed epithelial and stromal tumour represents a distinctive benign tumour of the kidney that should be distinguished from other cystic renal neoplasms. Prognosis of this tumour is favourable in nearly all cases published so far. Only rare cases of malignant transformation have been published. In summary, MEST represents a benign mostly cystic tumour of the kidney, which is predominately observed in middle aged, perimenopausal women. Knowledge of this certain but rare tumour entity is important, since in most cases conservative surgery with preservation of kidney function is the therapy of choice.

## Consent

Written informed consent was obtained from the patient for publication of this case report and any accompanying images. A copy of the written consent is available for review by the Editor-in-Chief of this journal.

## Competing interests

The authors declare that they have no competing interests.

## Authors' contributions

MR, JK and PM participated in conception of the idea and writing of the manuscript. WM and PM performed the histopathological interpretation of the tumour tissue.

## References

[B1] Michal M Syrucek M Benign mixed epithelial and stromal tumor of the kidney Pathol Res Pract 1998 194 445 448 9689654 10.1016/S0344-0338(98)80038-1

[B2] Turbiner J Amin MB Humphrey PA Srigley JR De LL Radhakrishnan A Oliva E Cystic nephroma and mixed epithelial and stromal tumor of kidney: a detailed clinicopathologic analysis of 34 cases and proposal for renal epithelial and stromal tumor (REST) as a unifying term Am J Surg Pathol 2007 31 489 500 10.1097/PAS.0b013e31802bdd56 17414095

[B3] Michal M Hes O Bisceglia M Simpson RH Spagnolo DV Parma A Boudova L Hora M Zachoval R Suster S Mixed epithelial and stromal tumors of the kidney. A report of 22 cases Virchows Arch 2004 445 359 367 10.1007/s00428-004-1060-y 15322873

[B4] Mohd Zam NA Lau WK Yip SK Cheng CW Tan PH Mixed epithelial and stromal tumour (MEST) of the kidney: a clinicopathological report of three cases Pathology 2009 41 403 406 10.1080/00313020902886944 19404861

[B5] Adsay NV Eble JN Srigley JR Jones EC Grignon DJ Mixed epithelial and stromal tumor of the kidney Am J Surg Pathol 2000 24 958 970 10.1097/00000478-200007000-00007 10895818

[B6] Montironi R Mazzucchelli R Lopez-Beltran A Martignoni G Cheng L Montorsi F Scarpelli M Cystic nephroma and mixed epithelial and stromal tumour of the kidney: opposite ends of the spectrum of the same entity? Eur Urol 2008 54 1237 1246 10.1016/j.eururo.2007.10.040 18006141

[B7] Antic T Perry KT Harrison K Zaytsev P Pins M Campbell SC Picken MM Mixed epithelial and stromal tumor of the kidney and cystic nephroma share overlapping features: reappraisal of 15 lesions Arch Pathol Lab Med 2006 130 80 85 16390243 10.5858/2006-130-80-MEASTO

[B8] Mai KT Elkeilani A Veinot JP Mixed epithelial and stromal tumour (MEST) of the kidney: report of 14 cases with male and PEComatous variants and proposed histopathogenesis Pathology 2007 39 235 240 10.1080/00313020701230799 17454754

[B9] Kuroda N Sakaida N Kinoshita H Matsuda T Hes O Michal M Okamoto S Nagashima Y Tanaka Y Carcinosarcoma arising in mixed epithelial and stromal tumor of the kidney APMIS 2008 116 1013 1015 10.1111/j.1600-0463.2008.01063.x 19133001

[B10] Nakagawa T Kanai Y Fujimoto H Kitamura H Furukawa H Maeda S Oyama T Takesaki T Hasegawa T Malignant mixed epithelial and stromal tumours of the kidney: a report of the first two cases with a fatal clinical outcome Histopathology 2004 44 302 304 10.1111/j.1365-2559.2004.01782.x 14987239

[B11] Svec A Hes O Michal M Zachoval R Malignant mixed epithelial and stromal tumor of the kidney Virchows Arch 2001 439 700 702 11764393 10.1007/s004280100518

[B12] Yap YS Coleman M Olver I Aggressive mixed epithelial-stromal tumour of the kidney treated with chemotherapy and radiotherapy Lancet Oncol 2004 5 747 749 10.1016/S1470-2045(04)01651-1 15581546

[B13] Eble JN Bonsib SM Extensively cystic renal neoplasms: cystic nephroma, cystic partially differentiated nephroblastoma, multilocular cystic renal cell carcinoma, and cystic hamartoma of renal pelvis Semin Diagn Pathol 1998 15 2 20 9503503

[B14] Lane BR Campbell SC Remer EM Fergany AF Williams SB Novick AC Weight CJ Magi-Galluzzi C Zhou M Adult cystic nephroma and mixed epithelial and stromal tumor of the kidney: clinical, radiographic, and pathologic characteristics Urology 2008 71 1142 1148 10.1016/j.urology.2007.11.106 18313107

[B15] Picken MM Fresco R Mixed epithelial and stromal tumor of the kidney: preliminary immunohistochemical and electron microscopic studies of the epithelial component Ultrastruct Pathol 2005 29 283 286 10.1080/01913120590951275 16036882

[B16] Beiko DT Nickel JC Boag AH Srigley JR Benign mixed epithelial stromal tumor of the kidney of possible mullerian origin J Urol 2001 166 1381 1382 10.1016/S0022-5347(05)65775-8 11547080

[B17] Algaba F Editorial comment on: cystic nephroma and mixed epithelial and stromal tumour of the kidney: opposite ends of the spectrum of the same entity? Eur Urol 2008 54 1245 1246 10.1016/j.eururo.2007.10.041 18006142

[B18] Hora M Michal M Hes O Re: Rodolfo Montironi, Roberta Mazzuccelli, Antonio Lopez-Beltran, et al. Cystic Nephroma and Mixed Epithelial and Stromal Tumour of the Kidney: Opposite Ends of the Spectrum of the Same Entity? Eur Urol 2008 54 1237 46 10.1016/j.eururo.2007.10.040 18006141

[B19] Tickoo SK Gopalan A Tu JJ Harik LR Al-Ahmadie HA Fine SW Olgac S Reuter VE Estrogen and progesterone-receptor-positive stroma as a non-tumorous proliferation in kidneys: a possible metaplastic response to obstruction Mod Pathol 2008 21 60 65 10.1038/modpathol.3800958 17873894

[B20] Zhou M Kort E Hoekstra P Westphal M Magi-Galluzzi C Sercia L Lane B Rini B Bukowski R Teh BT Adult cystic nephroma and mixed epithelial and stromal tumor of the kidney are the same disease entity: molecular and histologic evidence Am J Surg Pathol 2009 33 72 80 10.1097/PAS.0b013e3181be22d1 18971776

